# Metabolomic Diversity of Human Milk Cells over the Course of Lactation—A Preliminary Study

**DOI:** 10.3390/nu15051100

**Published:** 2023-02-22

**Authors:** Isabel Ten-Doménech, Mari Merce Cascant-Vilaplana, Víctor Navarro-Esteve, Birgit Felderer, Alba Moreno-Giménez, Iván Rienda, María Gormaz, Marta Moreno-Torres, David Pérez-Guaita, Guillermo Quintás, Julia Kuligowski

**Affiliations:** 1 Neonatal Research Group, Health Research Institute Hospital La Fe, Avda Fernando Abril Martorell 106, 46026 Valencia, Spain; 2 Department of Analytical Chemistry, University of Valencia, Dr. Moliner 50, 46100 Burjassot, Spain; 3 Master Program Biotechnical Processes, Austrian Biotech University of Applied Sciences, Konrad Lorenz-Strasse 10, 3430 Tulln, Austria; 4 Servicio de Anatomía Patológica, University & Polytechnic Hospital La Fe, Avda Fernando Abril Martorell 106, 46026 Valencia, Spain; 5 Division of Neonatology, University & Polytechnic Hospital La Fe, Avda Fernando Abril Martorell 106, 46026 Valencia, Spain; 6 Unidad de Hepatología Experimental y Trasplante Hepático, Health Research Institute Hospital La Fe, Avda Fernando Abril Martorell 106, 46026 Valencia, Spain; 7 Department of Biochemistry and Molecular Biology, University of Valencia, C/Blasco Ibáñez 15, 46010 Valencia, Spain; 8 Centro de Investigación Biomédica en Red de Enfermedades Hepáticas y Digestivas (CIBERehd), Instituto de Salud Carlos III, 28220 Madrid, Spain; 9 Health and Biomedicine, Leitat Technological Center, Carrer de la Innovació, 2, 08225 Terrassa, Spain

**Keywords:** human milk cells, metabolomics, infant nutrition, ultra-performance liquid chromatography–mass spectrometry (UPLC-MS), preterm infant

## Abstract

Human milk (HM) is a complex biofluid containing a wide cell variety including epithelial cells and leukocytes. However, the cellular compositions and their phenotypic properties over the course of lactation are poorly understood. The aim of this preliminary study was to characterize the cellular metabolome of HM over the course of lactation. Cells were isolated via centrifugation and the cellular fraction was characterized via cytomorphology and immunocytochemical staining. Cell metabolites were extracted and analyzed using ultra-performance liquid chromatography coupled to quadrupole time-of-flight mass spectrometry (UPLC–QqTOF-MS) in the positive and negative electrospray ionization modes. Immunocytochemical analysis revealed a high variability of the number of detected cells with relative median abundances of 98% of glandular epithelial cells, 1% of leukocytes, and 1% of keratinocytes. Significant correlations between the milk postnatal age with percentage of epithelial cells and leukocytes, and with total cell count were observed. Results from the Hierarchical Cluster Analysis of immunocytochemical profiles were very similar to those observed in the analysis of the metabolomic profiles. In addition, metabolic pathway analysis showed alterations in seven metabolic pathways correlating with postnatal age. This work paves the way for future investigations on changes in the metabolomic fraction of the cellular compartment of HM.

## 1. Introduction

Human milk (HM) is recognized as the gold standard for infant nutrition, offering numerous short and long-term benefits to the infant–mother dyad [1]. Especially for prematurely born infants, early nutrition has become a major player in improving clinical outcomes. Based on an impressive array of benefits, own mother’s milk (OMM) is the first choice for feeding preterm infants, and the selection of alternative means of feeding should be made in light of their effect on infant health [2]. While formula-fed infants show higher rates of weight gain and growth, the consumption of formula milk also increases the risk of necrotizing enterocolitis (NEC) [3]. On the other hand, increasing the intake of OMM and/or donor HM results in significant improvements in morbidity in the population of preterm infants [4].

The complex composition of HM is part of a dynamic system that responds to the needs of both infant and mother. In addition to the balanced content of essential nutrients, HM contains bioactive components such as growth and immunological factors, extracellular vesicles, and cells that are involved in remodeling the immune system and that alter infant susceptibility to infection. Literature reports present several metabolomic analyses of HM, including the study of associations of HM components with different clinical (e.g., gestational age, type of delivery) and environmental (e.g., geographical location, lifestyle) factors [5,6]. Furthermore, changes in the metabolome and lipidome across lactation [7] and upon pasteurization have been recently described [8]. Regarding the cellular fraction of HM, its composition is heterogeneous and comprises mainly epithelial cells (e.g., lactocytes and myoepithelial cells), immune cells (i.e., leukocytes), stem cells, and commensal bacteria. It has been observed that cell-type frequencies shift over the course of lactation, yielding greater epithelial diversity at later points [9]. The proportion of leukocytes found in HM also depends on the stage of lactation, with the cell count of leukocytes in colostrum being higher than in mature milk, where only approximately 2% of the total cells are leukocytes [10]. Although the presence of a variety of maternal leukocytes has been reported in both colostrum and mature HM [9], understanding the trafficking of these leukocytes and the significance of their transmission to the suckling offspring is still limited. In fact, controversial literature reports can be encountered with some reports demonstrating that milk-derived cells survive the harsh environment of the digestive tract and interact with the infant’s mucosal immune system in the intestine [11] and are even detectable in blood and brain of suckling pups [12], while others claim that gastric digestion induces apoptosis in the majority of cells present in HM indistinctive of the cell population [13]. The authors further suggest that although HM cells may not survive digestion, they still might be taken up by phagocytic cells or shed extracellular vesicles that can be taken up by the intestinal epithelium. At the same time, feeding interventions with cell-enriched HM in preterm infants with Bells’s stage I NEC have shown to successfully normalize fecal sphingolipids and the gut microbiome profiles resembling those of healthy controls [14]. In this regard, the role of metabolites of the HM cell fraction in achieving the benefits of breastfeeding remains so far unexplored. A better understanding of their roles could shed light on the functions of cells present in HM as well as provide actionable findings for future studies focused on the development of improved formulas and supplements to foster infant health.

The aim of this preliminary study was to provide insight into the HM cell metabolome and how it varies over the course of lactation. A pilot study was conducted involving fifteen HM samples collected at different postnatal ages. The cellular fraction was studied with microscopy and staining techniques, and their metabolic fingerprint was analyzed by ultra-performance liquid chromatography coupled to quadrupole time-of-flight mass spectrometry (UPLC–QqTOF-MS). To the best of our knowledge, the results presented are the first report on the metabolome of HM cells and we anticipate that they will foster further studies focusing on the role of HM cell contents in infant health, development, and nutrition.

## 2. Materials and Methods

### 2.1. Study Population and Sample Collection

Fifteen volunteers participating in this study were enrolled at the Human Milk Bank of the University & Polytechnic Hospital La Fe (Valencia, Spain). The sole inclusion criterion was the acceptance of the lactating mother to participate, after which they signed an informed consent form. Lactating mothers suffering from transmittable diseases were excluded.

Recruitment was carried out between November and December 2021. Demographic (maternal age, infant sex), anthropometric (birth weight, length, and head circumference) and clinical data (type of delivery, gestational age, postnatal age, APGAR scores at 1, 5, and 10 min) from the mothers and their infants were collected and are shown in Table S1. HM was collected as follows: hands were cleaned with water and soap for at least 15 s, followed by drying with a clean towel and sterilization of hands with hand sanitizer. Then, the skin area that comes into contact with the milk pump was cleaned with water and dried with sterile gauzes. No ointments were applied to the skin before HM extraction. Finally, the HM was extracted using a clean and sterilized breast milk pump and stored in clean sterile bottles. A 30 mL HM aliquot from full expression collected between 8 AM and 3 PM was set aside for immediate extraction of HM cells. Samples were labeled as colostrum (n = 1), transitional (n = 3) or mature (n = 11) milk according to postnatal age (i.e., 1–3 days (colostrum), 4–14 days (transitional), >14 days (mature)).

### 2.2. Cytochemical and Immunocytochemical Characterization of HM Cells

A 5-10 mL aliquot of fresh HM was centrifuged for 20 min at 800× *g* and 4 °C. The supernatant was discarded, and the pellet was resuspended in phosphate-buffered saline (PBS) (VWR, Barcelona, Spain) and centrifuged for 5 min at 800× *g* and 4 °C. The washing step was repeated. Finally, the recovered pellet was fixed by adding 0.25 mL PBS and 0.25 mL ThinPrep CytoLyt^®^ Solution (Hologic^®^, Marlborough, USA) and stored at room temperature prior to cytochemical and immunocytochemical characterization of cells. Three monolayers of cells were obtained from each cell suspension by centrifugation on a slide with Cytospin^®^ centrifuge (Thermo Scientific^®^, Waltham, USA) at 1100 rpm (137× *g*) for 12 min.

Cytochemical analysis was performed with the automatized Papanicolaou cytochemical stain (Sigma-Aldrich^®^, Merck^®^) with the Gemini AS^®^ Thermo Scientific^®^ slide stainer system. For the overall cell count per high power field (HPF) (40X/0.65, Zeiss A-Plan^®^, Zeiss Axiostar plus^®^), the identification of the field with maximum cellularity was selected. Immunocytochemical analysis was performed with (i) automatized CK AE1/AE3 (monoclonal mouse anti-human cytokeratin clone AE1/AE3) stain for characterization and counting of glandular epithelial cells per HPF; and (ii) CD45 (monoclonal mouse anti-human CD45 leukocyte common antigen clones 2B11 + PD7/26, Dako^®^) stain with Dako Omnis^®^ immunostainer for identification and counting of leukocytes.

Cell diversity within a sample was assessed using the Simpson’s Index of Diversity (1-*D*), where *D* was defined as:

(1) $$D=\frac{\sum n\left(n-1\right)}{N\left(N-1\right)}\;$$

where *n* is the number of cells of a particular cell type in the sample, and *N* is the total cell count. Thus, the higher the value for this index (1-*D*), the higher the cell diversity.

### 2.3. Metabolomic Fingerprinting of HM Cells

A 15 mL aliquot of fresh HM was centrifuged for 20 min at 800× *g* and 4 °C. The supernatant was discarded, and the pellet was resuspended in PBS and centrifuged for 5 min at 800× *g* and 4 °C. The washing step was repeated. For cell lysis and metabolite extraction, 300 µL of lysis solution (i.e., CH_3_OH:H_2_O 3:1, *v*/*v*) containing the internal standards (IS) L-tryptophan-D_5_ (≥99%), caffeine-D_9_ (≥99%), and L-phenylalanine-D_5_ (≥99%) at 0.25 μM each were added to the cell pellet. The mixture was transferred to a microcentrifuge tube placed on ice and 200 µL of the lysis solution was additionally employed to ensure quantitative transfer of cells. After three freeze–thaw cycles, samples were stored at −80 °C until further processing.

After thawing, the samples were centrifuged at 15,493× *g* and 4 °C for 5 min. The supernatant was transferred to a microcentrifuge tube and evaporated under vacuum at 30 °C on a miVac centrifugal vacuum concentrator (Genevac LTD, Ipswich, UK). Dry residues were resuspended in 70 µL of H_2_O:CH_3_CN (85:15 *v*/*v*, 0.1% *v*/*v* HCOOH) and homogenized, followed by a centrifugation at 15,493× *g* and 4 °C for 5 min to remove cell debris. Supernatants were transferred to microcentrifuge tubes and stored at −80 °C until analysis. A pooled quality control (QC) sample was prepared by mixing 10 µL of each sample extract. A blank extract was prepared following the same procedure as described for samples, but without cells. In addition, the supernatant obtained from the second washing step of one sample was processed as described for samples and analyzed.

Metabolomic analysis of HM cell extracts was carried out by UPLC–QqTOF-MS as described elsewhere [8]. Briefly, separations were performed on a Synergi^TM^ Hydro-RP 80 Å LC C_18_ column (150 × 2 mm, 4 μm, Phenomenex, Torrance, USA) in a 1290 Infinity UPLC system running the following binary gradient with solvent A (H_2_O, 0.1% *v*/*v* HCOOH) and solvent B (CH_3_CN, 0.1% *v*/*v* HCOOH) as mobile phase components: 1% B for 2 min, linear gradient from 1 to 80% B in 8 min, from 80 to 98% B in 0.1 min, 98% B for 1.9 min, return to initial conditions in 0.1 min, and column equilibration with 1% B for 2.9 min. The flow rate was set to 0.4 mL min^−1^ and column and autosampler temperatures were set at 40 and 4 °C, respectively.

For MS detection, an Agilent 6550 Spectrometer iFunnel quadrupole time-of-flight (QqTOF) MS system was used operating in ESI^+^ and ESI^-^ modes. Full scan MS data were acquired between 70 and 1500 *m*/*z* using the following ionization parameters: gas T, 200 °C; drying gas, 14 L min^−1^; nebulizer, 30 psi; sheath gas T, 350 °C; sheath gas flow, 10 L min^−1^. Mass reference standards were introduced into the source for automatic MS spectra recalibration during analysis via a reference sprayer valve using the 149.02332 (background contaminant), 121.050873 (purine), and 922.009798 (HP-0921) *m*/*z* as references in ESI^+^, and 119.036 (purine) and 980.0163 ([HP-0921 + CH_3_COOH-H]^−^) *m*/*z* in ESI^-^. MS^2^ data were acquired using iterated data dependent acquisition as described elsewhere [15] using centroid mode at a rate of 5 Hz in the extended dynamic range mode (2 GHz), a collision energy set to 20 V, medium isolation window (~4 amu), MS^2^ fragmentation with automated selection of five precursor ions per cycle, and an exclusion window of 0.15 min after two consecutive selections of the same precursor. UPLC-MS data acquisition was carried out employing MassHunter Workstation (version B.07.00) from Agilent.

The analytical batch included an initial system suitability check (2 μM IS solution) followed by 9 QC replicates for system conditioning and MS^2^ data acquisition. HM sample extracts were injected in random order. The QC was injected once at the beginning, twice at the end, and during the batch for the assessment of instrumental performance [16]. The blank extract was injected twice and the blank extract from cell supernatant once at the end of the measurement sequence to identify signals from origins other than biological origins, and possible carryover [17].

### 2.4. Data Processing and Analysis

Centroid UPLC-QqTOF-MS raw data were converted to mzXML format employing ProteoWizard [18] (http://proteowizard.sourceforge.net, accessed on 8 March 2022). XCMS software [19] and CAMERA [20] running in R 4.0.5 were employed for the generation of peak tables. The selection of parameters for peak table extraction and alignment for metabolomics was based on the observed variation of retention time (RT) and *m/z* values of ISs. The *centWave* method with the following settings was used for peak detection: *m/z* range = 70–1500, ppm = 15, peakwidth = (5 and 20), snthr = 6, prefilter = (3, 100). A minimum difference in *m/z* of 0.01 Da was selected for overlapping peaks. Intensity-weighted *m/z* values of each feature were calculated using the *wMean* function. Peak limits used for integration were found through descent on the Mexican hat filtered data. Peak grouping was carried out using the “density” method using mzwid = 0.015 and bw = 5. RT correction was carried out using the “obiwarp” method. After peak grouping, the fillPeaks method with the default parameters was applied to fill missing peak data. Automatic integration was assessed by comparison to manual integration using IS signals. A total of 1254 (ESI^+^) and 1615 (ESI^−^) features were initially detected after peak detection, integration, chromatographic deconvolution, and alignment in HM cell samples.

Further data processing and statistical analysis were carried out in MATLAB 2021a (Mathworks Inc., Natick, MA, USA) using in-house written scripts available from the authors and the PLS Toolbox 8.7 (Eigenvector Research Inc., Wenatchee, USA). Metabolites were annotated (identification level 2 [21]) by automatic matching of experimental MS/MS spectra to databases (i.e., Human Metabolome Data Base (HMDB) [22]) as described elsewhere [15] with the following parameters: *m*/*z* accuracy error between experimental and database parent ion, 20 ppm; difference in RT between parent ion and MS/MS spectra, 0.5 min; intensity threshold to remove low intensity MS/MS signals, absolute intensity <50 AU or relative intensity <0.01% of base peak; MS/MS spectra normalization, base peak; minimum number of ions in each experimental and database spectrum, 3; minimum number of matching ions between experimental and database spectrum, 3. In addition, LipiDex [23] was used for the annotation of lipids (*m*/*z* accuracy 20 ppm) using LipidBlast. Features for which the ratio between the median peak area values in QCs and blanks was lower than two were classified as unreliable and removed from further analysis. Intra-batch effect correction was performed using the Quality Control–Support Vector Regression algorithm employing a Radial Basis Function kernel [24] and the LIBSVM library [25] with the following parameters: ε–range = 2 to 5%; γ-range = 1 to 10^5^; C = 90%. Features with RSD (QC) > 20% after QC-SVRC were classified as unreliable and removed from further analysis. Thus, 149 (ESI^+^) and 166 (ESI^−^) features remained. Data were used without further normalization, as the aim of the study was to assess how the metabolome of the cellular fraction evolves along lactation to reveal aspects relevant with respect to the infant’s intake.

Unsupervised statistical methods, i.e., Hierarchical Cluster Analysis (HCA) and Principal Component Analysis (PCA) were performed using the PLS Toolbox in MATLAB 2021a and used to assess sample clustering. Pearson’s linear correlation coefficients were determined between continuous variables, i.e., metabolite abundances and the postnatal age, and the Wilcoxon rank sum test was used for between-group comparisons of clinical parameters (i.e., infant sex, type of delivery, and gestational age) with regard to the cell count. The false discovery rate (FDR) from the *p*-values of multiple-hypothesis testing was estimated using the procedure described by Benjamini and Hochberg [26], and adjusted *p*-values < 0.05 were considered statistically significant. Pathway analysis was performed using Metaboanalyst (version 5.0) [27] (www.metaboanalyst.ca, accessed on 7 February 2023 ) and the *Homo sapiens* pathway library of the Kyoto Encyclopedia of the Genes and Genomes (KEGG) database [28].

## 3. Results

### 3.1. Immunocytochemical Analysis

**Figure 1:**
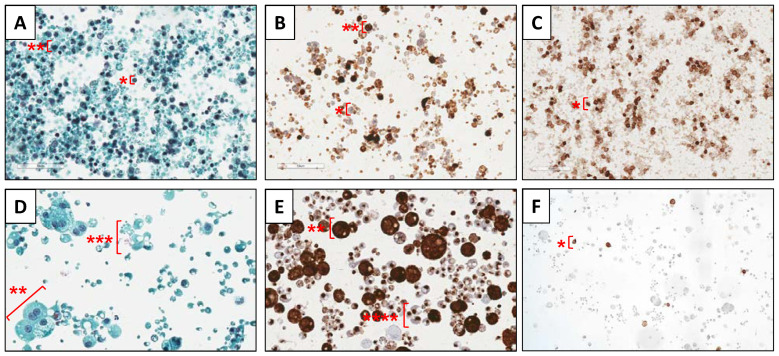
Cytochemical (Papanicolaou stain (**A**,**D**)) and immunocytochemical sections (CK AE/AE3 stain (**B**,**E**); CD45 stain (**C**,**F**)) of HM cells of colostrum (**A**–**C**) and mature HM (**D–F**). Note: *, leukocytes; **, glandular epithelial cells; ***, apocrine secretions of lipid globules.

Figure 1A,D show sections of Papanicolaou stains from the colostrum and one mature HM sample, respectively. The nucleus of epithelial cells and leukocytes can be seen in dark blue, while the cytoplasm is stained light blue. Leukocytes present smaller nucleus-to-cytoplasm ratio and size (from 5 to 10 µm approximately) than epithelial cells (from 10 to 25 µm approximately) and some binucleated cells can be observed in the latter cell type. Keratinocytes, with greater cell size, are stained orange (not shown in sections selected). Fatty macrovacuoles were identified as white vacuoles located inside the cells. Apocrine secretions of lipid globules were identified as extracellular light blue vesicles without a nucleus containing optically clear macro- and microvacuoles. Figure 1B,E show the immunocytochemical CK AE1/AE3 stain of the same samples, where the cytoplasm of glandular epithelial cells is stained in dark brown. Light brown cells with blue colored nucleus are assumed to be leukocytes (Figure 1B) and extracellular vesicles with cytokeratins aggregated and colored in dark brown were attributed to apocrine secretions (Figure 1E). Figure 1C,F show CD45 immunocytochemical stain in which positive reactivity for leukocytes can be observed in dark brown in the colostrum sample (Figure 1C), whereas positive cells arising in the mature milk sample are scarce (Figure 1F).

Table 1 provides cell counts of glandular epithelial cells, leukocytes, and keratinocytes in the set of HM samples analyzed. The colostrum sample was markedly different from transitional and mature HM samples based on the number of cells and %leukocytes.

**Figure 2:**
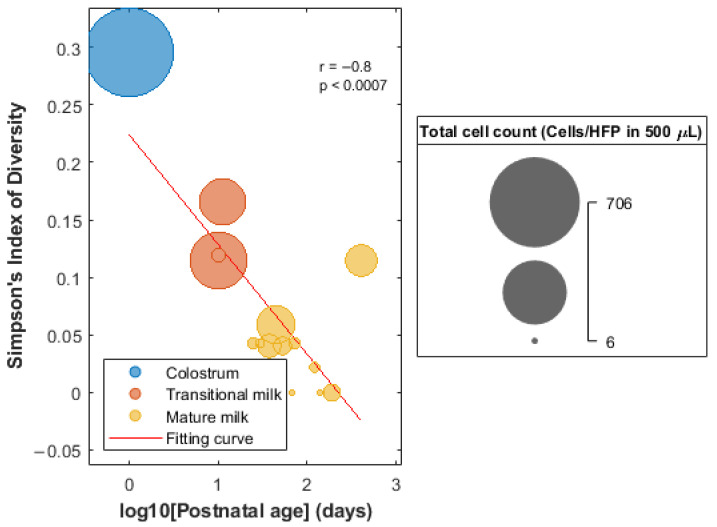
Evolution of the Simpson Index of Diversity of HM cells as a function of the postnatal age.

No significant differences (Wilcoxon ranksum test, *p*-value < 0.05) were found in the number of total cells or the proportions of cells of the individual cell types (i.e., epithelial cells, leukocytes, and keratinocytes) between mothers with male and female infants, spontaneous vaginal delivery and caesarean section, and term (i.e., gestational age ≥37 weeks) and preterm delivery (gestational age < 37 weeks). As shown in Figure S1, a significant, linear decrease in the overall cell count and proportion of leukocytes with postnatal age was observed, while the amount of glandular epithelial cells increased linearly along lactation (Pearson correlation, *p*-value < 0.05). No upward or downward trend of %keratinocytes with postnatal age was observed. Simpson Index of Diversity was calculated as a surrogate of cell diversity in HM samples and its correlation with postanal age was evaluated. Figure 2 shows a decrease in the cell count and diversity as a function of lactation age.

**Figure 3:**
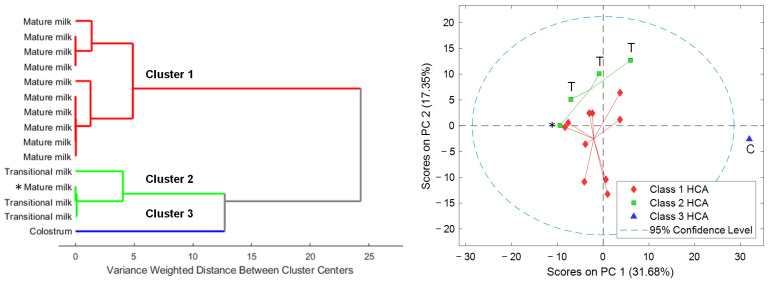
Dendrogram of the HCA (Ward’s method, 1 principal component) of the abundances of different cell types in the HM samples (**left**). PCA scores plot of the metabolomic fingerprints (**right**). Note: Classes 1 to 3 obtained from HCA of HM cell composition. C, colostrum; T, transitional milk; otherwise, mature milk.

HCA revealed three distinct clusters based on the number and proportion of the different cell types detected in HM samples (see Figure 3, left). Cluster 3 separated, as expected, the colostrum sample from the remaining samples according to the clear differences in total cell count and cell type composition in comparison to transitional and mature HM samples. On the other hand, cluster 2 included transitional milk samples and one mature milk sample, and cluster 1 gathered exclusively mature milk samples.

### 3.2. Metabolomic Analysis of HM Cells

Metabolomic analysis enabled the detection of 315 features in the cell extracts. Initially, PCA was carried out to identify the main sources of variation in the retrieved metabolic profiles. Scatter plots of PCA scores shown in Figure 3 (right) showed a sample distribution in the scores space that matched the clustering previously observed based on their cytochemical profiles. Furthermore, results showed a clustering of samples according to lactation stage (i.e., colostrum, transitional milk, mature milk). It has to be noted that the mature milk sample included in cluster 2 based on immunocytochemical data (Figure 3, left, highlighted with an asterisk) was closer to the mature milk samples grouped in the PC1-PC2 scores space (see Figure 3, right) from the analysis of the metabolic profiles.

**Figure 4:**
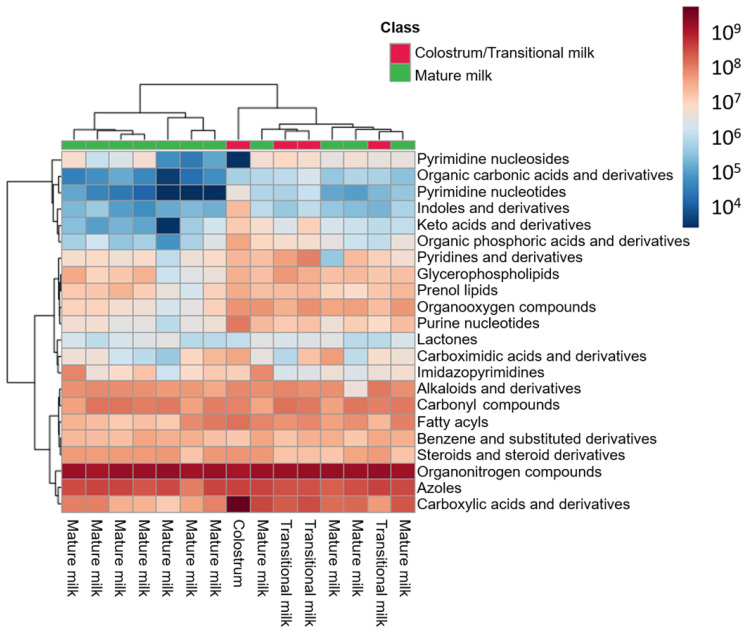
Heatmap of metabolite abundance grouped by metabolite class in HM cells.

Among the total number of features, 66 (i.e., 21%) were annotated based upon their spectral similarity with HMDB and LipidBlast. The correlation between the experimental and reference MS/MS spectra in terms of *m/z* values and intensities was assessed through the calculation of the mean dot product [23] (see Figure S2). After manual data curation, 63 features were considered metabolite-related features (see Table S2 and Figure S3, left): 22% of the annotated features belonged to the class of ‘carboxylic acids and derivatives’; 14% to the ‘organonitrogen compounds’; 13% to the ‘organooxygen compounds’; and 8% to ‘fatty acyls’ (see Figure S3, right). These features corresponded to 53 unique metabolites. Figure 4 shows the abundance of these unique metabolites grouped by class. As can be seen, the highest abundance in all samples was ascribed to the ‘organonitrogen compounds’ class. Within this class, phytosphingosine and sphinganine d16:0 (i.e., dihydrosphingosine d16:0; SP d16:0), two amines related with sphingolipid metabolism, are outstanding. Regarding the colostrum sample, higher abundances for all metabolite classes, and particularly for ‘carboxylic acids and derivatives’, compared to transitional and mature milk samples were observed, with the exception of metabolites from the ‘pyrimidine nucleosides’ class. The amino acid L-leucine was the most abundant metabolite ascribed to the former, whereas the free nucleoside cytidine, directly involved in pyrimidine metabolism, was the unique metabolite encountered in the latter.

**Figure 5:**
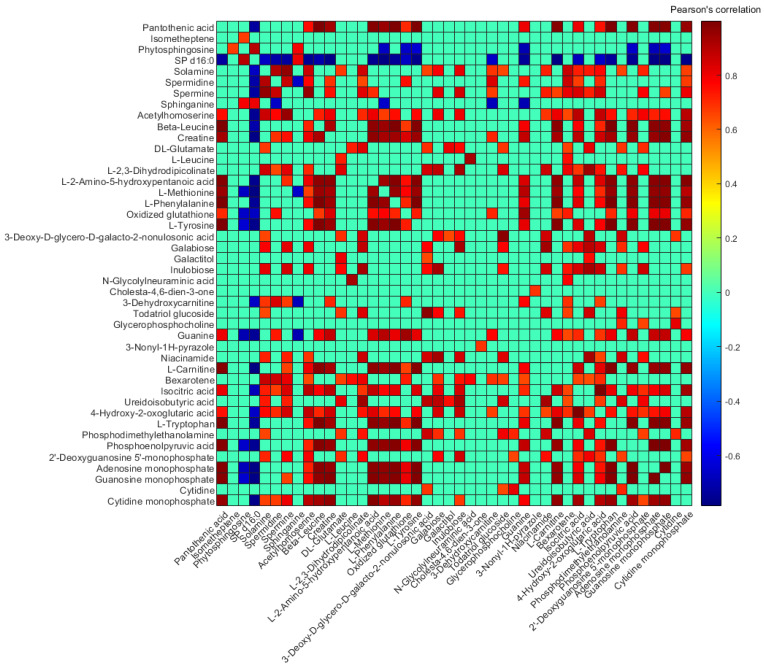
Pearson’s paired correlation between unique metabolites. Note: For better visualization, those correlations considered non-significant (*p*-value < 0.01) are given an r-value = 0, and those metabolites that did not correlate significantly with any other metabolite are not shown.

Figure 5 shows Pearson’s paired correlation among metabolites. Significant positive correlations were observed for most metabolites, and particularly between amino acids such as L-tyrosine, L-phenylalanine, L-methionine, and L-2-amino-5-hydroxypentanoic acid (5-hydroxy norvaline). Conversely, for SP d16:0, all significant correlations with other metabolites were negative, with the exception of its structurally related metabolite (i.e., sphinganine or dihydrosphingosine d18:0), and with phytosphingosine. Even though the high concentrations of certain metabolites in the colostrum sample skewed the computed correlations, Pearson’s paired correlation among metabolites excluding this sample from the analysis provided similar results (see Figure S4). Most of the significant positive correlations as well as the negative correlation of SP d16:0 with certain metabolites (i.e., L-tryptophan, L-carnitine, L-tyrosine, L-phenylalanine, spermine, spermidine, and solamine) were also observed.

Statistical analysis also showed significant linear correlations between metabolite abundancies and postnatal age (log-transformed days): 21 (i.e., 40%) metabolites were negatively correlated with postnatal age, and only SP d16:0 increased along lactation (Pearson’s correlation, adjusted *p*-value < 0.05). Amino acids and analogues such as acetylhomoserine, L-leucine, creatine, L-2-amino-5-hydroxypentanoic acid, L-methionine, L-phenylalanine, and L-tyrosine, amines such as solamine, spermidine, and spermine, and purine ribonucleotides such as adenosine monophosphate (AMP) and guanosine monophosphate (GMP) decreased with increasing postnatal age. Interestingly, we found a diacetylated derivative of spermine (i.e., N^1^,N^12^-diacetylspermine, DiAcSpm) that also decreased along lactation (r-value = −0.3), although not significantly (adjusted *p*-value > 0.05). Pathway analysis using the levels of these metabolites correlating with postnatal age (see Table S2) led to the identification of nine altered metabolic pathways (see Figure S5 and Table 2), five of which were related either with amino acid metabolism (i.e., ‘arginine and proline metabolism’, ‘phenylalanine, tyrosine and tryptophan biosynthesis’, and ‘phenylalanine metabolism’) or with the metabolism of other amino acids (i.e., ‘beta-alanine metabolism’ and ‘glutathione metabolism’).

## 4. Discussion

This is the first descriptive study reporting the metabolome of cells isolated from HM samples at different lactation stages, from colostrum to mature milk, with samples collected over more than one year after delivery (see Table S1). Isolated cells were characterized via immunocytochemical analysis identifying primarily glandular epithelial cells and, to a lesser extent, leukocytes (immune cells) and keratinocytes, which are the primary type of cells found in the epidermis (see Figure 1 and Table 1). These results were in agreement with previous reports aiming at the characterization of the cellular fraction of HM samples [9,29]. Leukocyte concentration in colostrum was found to be substantially greater than that in transitional milk and mature milk and did not significantly differ from one another in transitional milk and mature milk [30].

We observed a significant decrease in the relative leukocyte proportion and the total cell count with time and a concurrent relative increase in glandular epithelial cells (see Figure S1). Hassiotou et al. [31] observed that, under healthy conditions of the infant–mother dyad, colostrum always contained higher number of cells per mL of milk with considerable levels of leukocytes. However, leukocytes decreased rapidly during the first week postpartum, thus reaching low baseline levels from then on (i.e., 0–2%). These changes in cell count and diversity along lactation are presented in Figure 2, where the colostrum sample differs clearly from transitional and mature HM samples [9]. It should be noted that, in this study, previously reported patterns regarding cell count and diversity with postnatal age could be observed, even though samples from the different lactation stages were not collected from the same participants. In addition, considering only cell type distribution within the studied HM samples, three distinct sub-clusters, with one cluster containing the colostrum sample and the other two clusters revealing two sub-sets among the remaining transitional and mature HM cell samples could be differentiated (see Figure 3, left). This fact suggests that variability between individuals at the cellular level needs to be characterized in terms of cellular sub-populations among the different main cell types [28]. On the other hand, the determined cellular composition of HM samples did not show significant differences between infant sex, type of delivery, and gestational age (term vs. preterm). However, this study was not designed specifically to carry out these comparisons. There might be different confounding factors that affect the cellular composition of HM simultaneously. A study with a dedicated design with a larger sample size for elucidating alterations in HM cells with, e.g., gestational age is therefore pending assignments for the future.

Metabolomic analysis of cell lysates identified ‘carboxylic acids and derivatives’, ‘organonitrogen compounds’, ‘organooxygen compounds’, and ‘fatty acyls’ among other metabolite classes (see Figure 4 and Table S2) [32,33]. The classes of the annotated metabolites showed a minor overlap with results obtained from the analysis of the HM samples including the amino acids L-tryptophan, L-phenylalanine, L-methionine, and the disaccharide inulobiose [8]. However, these annotations (identification level 2 [21]) should be handled with caution and, if possible, confirmed with analytical standards to increase confidence. Remarkably, the pattern observed in the clustering of HM samples according to cell diversity could be projected onto the metabolic fingerprints recorded from the cells reflecting a similar separation into the different sub-clusters (see Figure 3, right). Hence, the three sub-clusters did not only show different compositions of determined cellular fractions, but accordingly also showed detectable differences in their metabolic composition. All HM cell fractions, with the exception of that derived from the colostrum sample, presented similar class abundances, and remarkably a high abundance of ‘organonitrogen compounds’ (see Figure 4). In particular, two amines related with sphingolipid metabolism (i.e., phytosphingosine and SP d16:0) stand out, with the latter increasing significantly proportional to postnatal age. These metabolites have been described as cell death mediators, since they exert antiproliferative and pro-apoptotic effects [34,35], which might be associated with the reduction in the total cell count observed along lactation.

Regarding the colostrum sample, overall, higher abundances for all metabolite classes and in particular for certain metabolites (e.g., L-leucine) compared to transitional and mature milk samples were observed. However, statistical comparisons between the groups were not performed due to the small sample size involved.

Significant correlations between metabolites within the cell fraction of the HM samples were observed (Figure 5 and Figure S4). Furthermore, when the lactation stage was considered as a continuous variable (i.e., postnatal age in days), nine metabolic pathways were altered across the time span considered (see Figure S5 and Table 2), five of them related either with amino acid metabolism or the metabolism of other amino acids (i.e., ‘arginine and proline metabolism’, ‘phenylalanine, tyrosine and tryptophan biosynthesis’, ‘phenylalanine metabolism’, ‘beta-alanine metabolism’, and ‘glutathione metabolism’). Amino acid homeostasis in mammalian cells is maintained thanks to the coordinated action of amino acid metabolism and transport [36]. Here, essential amino acids such as L-leucine, L-phenylalanine, L-methionine, and L-tryptophan were present in HM cell extracts, and they all decreased along lactation. Interestingly, the content of these amino acids in HM decreases as lactation progresses [37].

On the other hand, polyamines such as spermine and spermidine appeared as changing metabolites with lactation stage (downward trend), being involved in several altered pathways (see Table 2). These small biomolecules are crucial in many biological functions such as cell growth and proliferation, gene regulation or nucleic acid stabilization. The intracellular levels of these polyamines are primarily regulated via de novo synthesis. In this sense, HM supplies the infant with the first exogenous source of polyamines [38]. On the other hand, we tentatively identified DiAcSpm in our HM cell extracts. DiAcSpm is a minor polyamine component of human urine [39], but its presence has been also reported in other biofluids. In addition, its role in cancer development has been widely discussed in recent years [40]. In an untargeted metabolomic study, Liu et al. [41] reported significantly higher levels of DiAcSpm in the amniotic fluid of Trisomy 21 pregnancies compared to normal pregnancy amniotic fluid, although its potential influence on fetal development was not addressed by the authors. On the other hand, maternal serum levels of DiAcSpm differ by fetal sex and pregnancy complications such as preeclampsia and fetal growth restriction [42,43]. However, to our knowledge, this is the first study reporting the presence of DiAcSpm in HM cells.

The current study was designed as a proof-of-concept study and hence it has several limitations regarding the experimental design. The number of study participants and samples from different lactation stages (i.e., colostrum, transitional milk, mature milk) are small and no repeated sampling from the same donor was performed, limiting the possibility of discerning between confounding factors such as interindividual variability, gestational age, and lactation stage. The need to use fresh HM samples without freezing steps prior to cell isolation was a major obstacle jointly with the relatively high volume required for both metabolomics analysis and microscopic and staining techniques, especially regarding the collection of colostrum samples. These limiting factors conditioned especially the acquisition of additional colostrum samples in this study, since any interference in the mother–infant feeding practice must be avoided. Furthermore, the use of alternative methods (e.g., flow cytometry) would allow one to obtain more detailed information on the composition of the cellular fraction. Based on this, further comparative studies regarding the metabolic profiles in this type of sample could be addressed.

It should be noted that, in this study no post-acquisition normalization strategy, statistical (e.g., total abundance of each metabolic feature, median fold change) or chemical base (e.g., total cell count, total protein content) [44], was applied. While it is true that normalization aims to decrease unwanted analytical and biological variation, the aim of this study was not to compare the metabolic make-up of the cell fraction between individuals, but to show how this fraction in its entirety evolves along lactation, which might be more relevant with respect to the infant’s intake. In this sense, normalization strategies on metabolic profiles in this type of sample and their impact are encouraged in future studies.

HM is a food source that has specifically evolved over millions of years to perfectly meet the dynamic nutritional needs of infants, and yet, much remains to be discovered about its compositional changes in the different stages of lactation. The present work provides a preliminary longitudinal picture of the cellular fraction observed in HM samples spanning the time window from birth and until over one year of lactation, their metabolome and the metabolic pathways involved, as well as their associations with clinical maternal–infant meta-data. This work paves the way for future investigations to enhance our understanding of HM cells and their metabolic composition that could provide actionable findings in the field of infant nutrition or aid in the development of new preventive measures for health hazards and diseases (e.g., NEC), especially relevant for preterm infants.

## Figures and Tables

**Table 1:** Total cell count and percentage of cell types determined in HM samples.

	Colostrum	Transitional Milk	Mature Milk
Total cell count (cells/HPF in 500 μL)	706	196 (137)	16 (30)
Glandular epithelial cells (%)	82	94 (2)	98 (2)
Leukocytes (%)	18	5 (4)	1 (1)
Keratinocytes (%)	0	1 (2)	1 (1)

Note: Values represent median (IQR, interquartile range).

**Table 2:** Metabolic pathways altered with postnatal age.

Pathway	Class	*p*-Value	FDR	Impact	Total Compounds	Significant Hits	Metabolites
Purine metabolism	Nucleotide metabolism	0.0005	0.006	0.13	65	3	AMP, GMP, dGMP
Arginine and proline metabolism	Amino acid metabolism	0.001	0.006	0.05	38	3	Creatine, Spermidine, Spermine
Citrate cycle (TCA cycle)	Carbohydrate metabolism	0.003	0.006	0.04	20	2	Isocitrate, Phosphoenolpyruvate
Phenylalanine, tyrosine and tryptophan biosynthesis	Amino acid metabolism	0.003	0.006	1.0	4	2	L-Phenylalanine, L-Tyrosine
Phenylalanine metabolism	Amino acid metabolism	0.003	0.006	0.4	10	2	L-Phenylalanine, L-Tyrosine
Beta-Alanine metabolism	Metabolism of other amino acids	0.003	0.006	0.06	21	2	Spermine, Spermidine
Glutathione metabolism	Metabolism of other amino acids	0.003	0.006	0.007	28	2	Spermidine, Spermine
Glyoxylate and dicarboxylate metabolism	Carbohydrate metabolism	0.004	0.006	0.08	32	2	4-Hydroxy-2-oxoglutarate, Isocitrate
Aminoacyl-tRNA biosynthesis	Translation	0.004	0.006	0	48	5	L-Phenylalanine, L-Methionine, L-Leucine, L-Tryptophan, L-Tyrosine

Note: Pathways with <2 significant hits were removed from the results.

## Data Availability

MS^1^ and MS^2^ data and metadata are accessible via the Zenodo repository (https://zenodo.org/, accessed on 5 December 2022) under doi: 10.5281/zenodo.7398164.

## Supplementary Materials

The following supporting information can be downloaded at: https://www.mdpi.com/article/10.3390/nu15051100/s1, Table S1. Demographic, anthropometric, and clinical descriptors of the study population; Figure S1. Total cell count (A), % glandular epithelial cells (B), % leukocytes (C), and % keratinocytes (D) versus postnatal age. Note: postnatal age was log10 scaled; solid lines are regression lines and r stands for the Pearson correlation coefficient; Figure S2. Similarity match of LC-MS features (i.e., *m*/*z*-retention time) with the HMDB of selected metabolites in ESI+/ESI−. Note: MS/MS acquired (A) spectrum (up); MS/MS reference (R) spectrum (down). Red dots represent matched ions (i.e., ions detected in both A and R spectra within a 20 ppm tolerance); green dots show *m*/*z* value of parent ions in the A and R spectra; dot product value considers all ions for its calculation, while reverse dot product A&R and reverse dot product R consider only ions present both in A and R spectra, and only ions present in the R spectrum, respectively; Table S2. Characteristics of annotated LC-MS features; Figure S3. Annotated metabolic features in HM cells. Distribution of features in the *m*/*z* vs. retention time space (left) and proportion of annotated metabolite classes (right) detected in HM cells; Figure S4. Pearson’s paired correlation between unique metabolites. Note: For better visualization, those correlations considered non-significant (*p*-value < 0.05) are given an r-value = 0, and those metabolites that did not correlate significantly with any other metabolite are not shown; Figure S5. Pathway enrichment analysis results of metabolites correlating significantly with postnatal age.
