# Supplementary material for: Metabolomic Diversity of Human Milk Cells over the Course of Lactation—A Preliminary Study
Source: Nutrients. 2023 Feb 22;15(5):1100. doi: 10.3390/nu15051100 (PMC10005050; doi:10.3390/nu15051100)
Supplement: Supplementary file 1 [file nutrients-15-01100-s001.zip › nutrients-2182153-supplementary.pdf]

Supplementary Information

Metabolomic Diversity of Human Milk Cells over the Course of Lactation. A Preliminary Study

Isabel Ten-Doménech[1†], Mari Merce Cascant-Vilaplana[1†], Víctor Navarro-Esteve[1,2], Birgit Felderer[1,3], Alba Moreno-Giménez[1], Iván Rienda[4], María Gormaz[1,5], Marta Moreno-Torres[6,7,8], David Pérez-Guaita[2], Guillermo Quintás[9], Julia Kuligowski[1]*

[1]*Neonatal Research Group, Health Research Institute Hospital La Fe, Avda Fernando Abril Martorell 106, 46026 Valencia, Spain*

[2]*Department of Analytical Chemistry, University of Valencia, Dr. Moliner 50, 46100 Burjassot, Spain*

[3]*Austrian Biotech University of Applied Sciences, Konrad Lorenz-Strasse 10, 3430 Tulln, Austria*

[4]*Servicio de Anatomía Patológica, University & Polytechnic Hospital La Fe, Avda Fernando Abril Martorell 106, 46026 Valencia, Spain*

[5]*Division of Neonatology, University & Polytechnic Hospital La Fe, Avda Fernando Abril Martorell 106, 46026 Valencia, Spain*

[6]*Unidad de Hepatología Experimental y Trasplante Hepático, Health Research Institute Hospital La Fe, Avda Fernando Abril Martorell 106, 46026 Valencia, Spain*

[7]*Departament of Biochemistry and Molecular Biology, University of Valencia, C/ Blasco Ibáñez 15, 46010 Valencia, Spain*

[8]*Centro de Investigación Biomédica en Red de Enfermedades Hepáticas y Digestivas (CIBERehd), Instituto de Salud Carlos III, Madrid, Spain*

[9]*Health and Biomedicine, Leitat Technological Center, Carrer de la Innovació, 2, 08225 Terrassa, Spain*

[†]These authors contributed equally to this work

*Corresponding author: Julia Kuligowski, e-mail: julia.kuligowski@uv.es; Phone: +34/961246661

Table S1. Demographic, anthropometric, and clinical descriptors of the study population.

Parameters	Median (IQR) / N%	Min. - max. value
Mother's age [years], median (IQR)	37 (4)	28 - 39
Gestational age [weeks], median (IQR)	39 (4)	26 - 41
Birthweight [g], median (IQR)	3150 (1764)	800 - 3990
Length [cm], median (IQR)	50 (3)	41 - 53
Head circumference [cm], median (IQR)	34 (7)	26 - 37
Spontaneous vaginal delivery [%], N	29	n.d.
Male infants [%], N	79	n.d.
Apgar 1, median (IQR)	9 (4)	1 - 10
Apgar 5, median (IQR)	10 (1)	2 - 10
Apgar 10, median (IQR)	10 (1)	8 - 10
Postnatal age [days], median (IQR)	45 (80)	1 - 402

Note: IQR, interquartile range

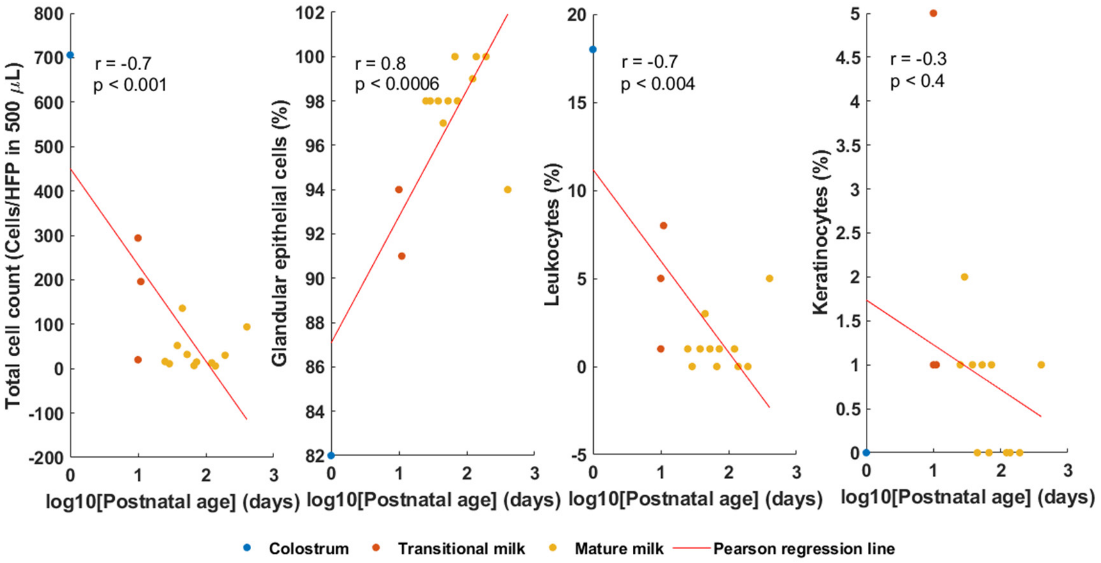

Figure S1. Total cell count (A), % glandular epithelial cells (B), % leukocytes (C), and % keratinocytes (D) versus postnatal age. Note: postnatal age was log10 scaled; solid lines are regression lines and r stands for the Pearson correlation coefficient.

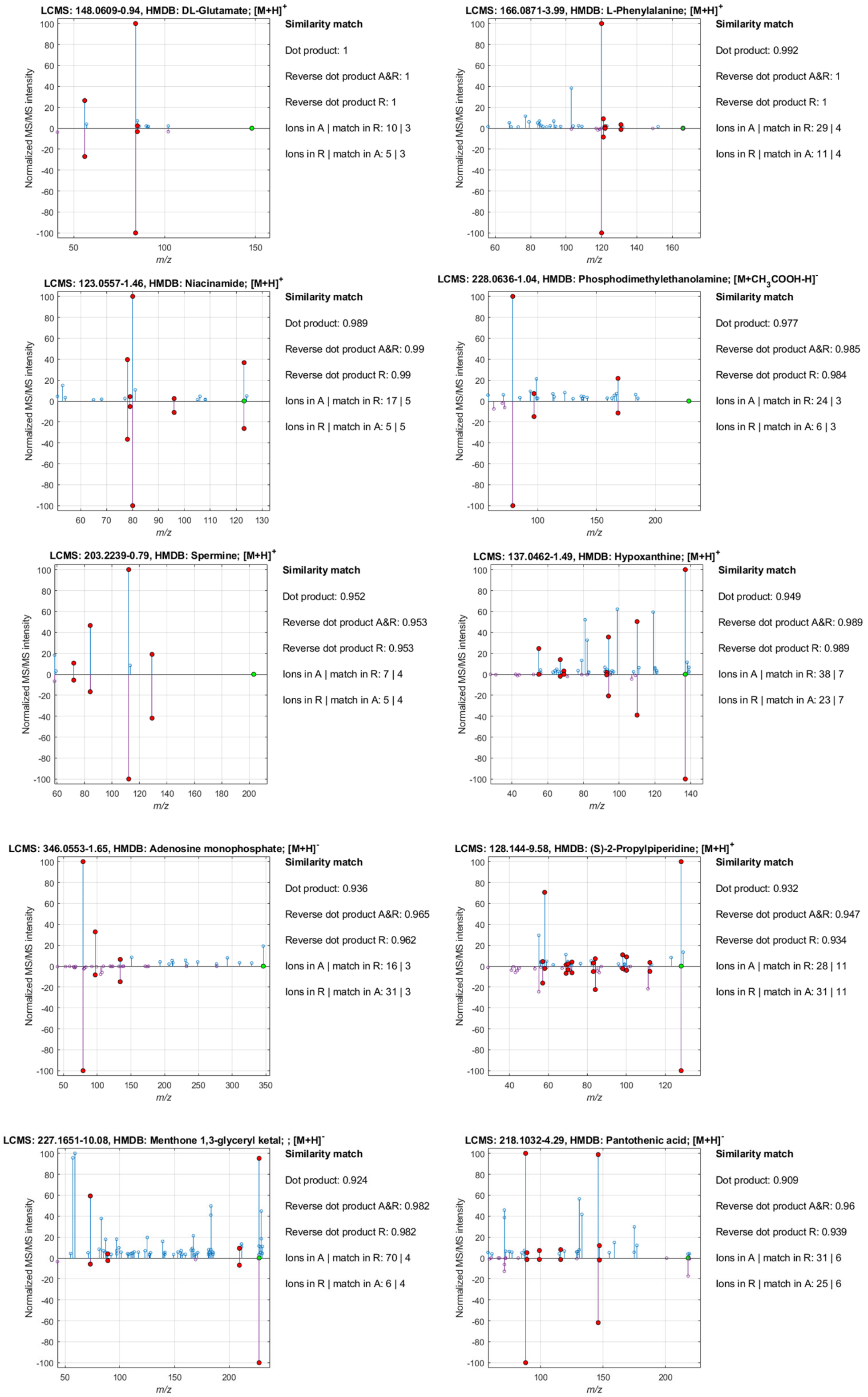

Figure S2. Similarity match of LC-MS features (i.e., *m/z*-retention time) with the HMDB of selected metabolites in ESI^+/ESI^-. Note: MS/MS acquired (A) spectrum (up); MS/MS reference (R) spectrum (down). Red dots represent matched ions (i.e., ions detected in both A and R spectra within a 20 ppm tolerance); green dots show *m/z* value of parent ions in the A and R spectra; dot product value considers all ions for its calculation, while reverse dot product A&R and reverse dot product R consider only ions present both in A and R spectra, and only ions present in the R spectrum, respectively.

Table S2. Characteristics of annotated LC-MS features.

#No.	Metabolite	RT (min)	*m/z*	Adduct	No. MS/MS ions matched	dp	rdp	mean dp	Class	Subclass	KEGG ID	HMDB
#1	Pyrimethanil	0.22	109.076	$[M+H+NH_4]^{2+}$	4	0.15	0.94	0.37	Benzene and substituted derivatives	Aniline and substituted anilines	C11180	HMDB0033135
**#2**	**Spermidine**	**0.75**	**146.166**	$[M+H]^+$	**3**	**0.62**	**0.96**	**0.77**	**Organonitrogen compounds**	**Amines**	**C00315**	**HMDB0001257**
**#3**	**Solamine**	**0.76**	**129.139**	$[M+2H+ACN]^{2+}$	**5**	**0.15**	**0.56**	**0.29**	**Organonitrogen compounds**	**Amines**	**NA**	**HMDB0031202**
#4	*Solamine	0.76	112.112	-	-	-	-	-	Organonitrogen compounds	Amines	-	-
**#5**	**Spermine**	**0.79**	**203.224**	$[M+H]^+$	**4**	**0.95**	**0.95**	**0.95**	**Organonitrogen compounds**	**Amines**	**C00750**	**HMDB0001256**
#6	Ureidoisobutyric acid	0.87	145.061	$[M-H]^-$	3	0.85	0.99	0.92	Organic carbonic acids and derivatives	Ureas	C05100	HMDB0002031
#7	*L-2,3-Dihydrodipicolinate	0.88	168.028	-	-	-	-	-	Carboxylic acids and derivatives	Amino acids, peptides, and analogues	-	-
#8	L-2,3-Dihydrodipicolinate	0.89	214.035	$[M+HCOOH-H]^-$	3	0.40	0.91	0.61	Carboxylic acids and derivatives	Amino acids, peptides, and analogues	C03340	HMDB0012247
#9	*Inulobiose	0.89	179.056	-	-	-	-	-	Organooxygen compounds	Carbohydrates and carbohydrate conjugates	-	-
#10	Todatriol glucoside	0.89	409.097	$[M+K-2H-H_2O]^-$	4	0.21	0.97	0.45	Fatty Acyls	Fatty acyl glycosides	NA	HMDB0037260
#11	3-Deoxy-D-glycero-D-galacto-2-nonulosonic acid	0.89	289.054	$[M+Na-2H]^-$	5	0.43	0.61	0.51	Organooxygen compounds	Carbohydrates and carbohydrate conjugates	NA	HMDB0000425
**#12**	**Inulobiose**	**0.89**	**387.115**	$[M+HCOOH-H]^-$	**6**	**0.77**	**0.89**	**0.83**	**Organooxygen compounds**	**Carbohydrates and carbohydrate conjugates**	**C03323**	**HMDB0029898**
#13	*3-Deoxy-D-glycero-D-galacto-2-nonulosonic acid; *Inulobiose; *Todatriol glucoside	0.89	161.045	-	-	-	-	-	Organooxygen compounds	Carbohydrates and carbohydrate conjugates	-	-
#14	Galactitol	0.90	181.072	$[M-H]^-$	3	0.75	0.96	0.85	Organooxygen compounds	Carbohydrates and carbohydrate conjugates	C01697	HMDB0000107
#15	Galabiose	0.92	341.109	$[M-H]^-$	13	0.59	0.83	0.70	Organooxygen compounds	Carbohydrates and carbohydrate conjugates	C00760	HMDB0029902
#16	*Cytidine	0.92	242.080	-	-	-	-	-	Pyrimidine nucleosides	Pyrimidine nucleosides	-	-
#17	Cytidine	0.92	302.101	$[M+CH_3COOH-H]^-$	3	0.84	0.90	0.87	Pyrimidine nucleosides	Pyrimidine nucleosides	C00475	HMDB0000089
#18	DL-Glutamate	0.94	148.061	$[M+H]^+$	3	1.00	1.00	1.00	Carboxylic acids and derivatives	Amino acids, peptides, and analogues	C00302	HMDB0060475
**#19**	**L-Carnitine**	**0.95**	**162.113**	$[M+H]^+$	**5**	**0.75**	**0.80**	**0.77**	**Organonitrogen compounds**	**Quaternary ammonium salts**	**C00318**	**HMDB0000062**
#20	Glycerophosphocholine	0.98	258.111	$[M+H]^+$	5	0.10	0.69	0.27	Glycerophospholipids	Glycerophosphocholines	C00670	HMDB0000086
**#21**	**Creatine**	**0.98**	**132.077**	$[M+H]^+$	**3**	**0.58**	**0.68**	**0.63**	**Carboxylic acids and derivatives**	**Amino acids, peptides, and analogues**	**C00300**	**HMDB0000064**
#22	N^1,N^{12}-Diacetylspermine	0.99	287.245	$[M+H]^+$	6	0.53	0.97	0.71	Carboximidic acids and derivatives	Carboximidic acids	C03413	HMDB0002172
#23	*4-Hydroxy-2-oxoglutaric acid;*Isocitric acid	1.00	71.014	-	-	-	-	-	Keto acids and derivatives	Gamma-keto acids and derivatives	-	-
**#24**	**L-2-Amino-5-hydroxypentanoic acid**	**1.01**	**116.071**	$[M+H-H_2O]^+$	**3**	**0.67**	**0.76**	**0.71**	**Carboxylic acids and derivatives**	**Amino acids, peptides, and analogues**	**NA**	**HMDB0031658**
**#25**	**Isocitric acid**	**1.01**	**173.009**	$[M-H-H_2O]^-$	**3**	**0.22**	**0.75**	**0.41**	**Carboxylic acids and derivatives**	**Tricarboxylic acids and derivatives**	**C00311**	**HMDB0000193**
**#26**	**Phosphoenolpyruvic acid**	**1.01**	**226.996**	$[M+CH_3COOH-H]^-$	**3**	**0.69**	**1.00**	**0.83**	**Organic phosphoric acids and derivatives**	**Phosphate esters**	**C00074**	**HMDB0000263**
**#27**	**4-Hydroxy-2-oxoglutaric acid**	**1.02**	**142.998**	$[M-H-H_2O]^-$	**4**	**0.32**	**0.76**	**0.50**	**Keto acids and derivatives**	**Gamma-keto acids and derivatives**	**C01127**	**HMDB0002070**
#28	Phosphodimethylethanolamine	1.04	228.064	$[M+CH_3COOH-H]^-$	3	0.98	0.98	0.98	Organic phosphoric acids and derivatives	Phosphate esters	C13482	HMDB0060244
#29	3-Dehydroxycarnitine	1.04	184.074	$[M+K]^+$	3	0.20	0.61	0.35	Fatty Acyls	Fatty acids and conjugates	C05543	HMDB0006831
**#30**	**Cytidine monophosphate**	**1.07**	**322.044**	$[M-H]^-$	**3**	**0.68**	**0.72**	**0.70**	**Pyrimidine nucleotides**	**Pyrimidine ribonucleotides**	**C00055**	**HMDB0000095**
#31	3-Dehydroxycarnitine	1.13	184.074	$[M+K]^+$	3	0.21	0.64	0.37	Fatty Acyls	Fatty acids and conjugates	C05543	HMDB0006831
**#32**	**Acetylhomoserine**	**1.17**	**162.077**	$[M+H]^+$	**3**	**0.57**	**1.00**	**0.76**	**Carboxylic acids and derivatives**	**Amino acids, peptides, and analogues**	**C01077**	**HMDB0029423**

#No.	Metabolite	RT (min)	*m/z*	Adduct	No. MS/MS ions matched	dp	rdp	mean dp	Class	Subclass	KEGG ID	HMDB
#33	Guanine	1.36	152.057	$[M+H]^+$	6	0.72	0.90	0.81	Imidazopyrimidines	Purines and purine derivatives	C00242	HMDB0000132
**#34**	**L-Methionine**	**1.39**	**150.059**	**$[M+H]^+$**	**6**	**0.42**	**0.64**	**0.52**	**Carboxylic acids and derivatives**	**Amino acids, peptides, and analogues**	**C00073**	**HMDB0000696**
#35	Glutathione	1.42	308.093	$[M+H]^+$	4	0.09	0.94	0.29	Carboxylic acids and derivatives	Amino acids, peptides, and analogues	C00051	HMDB0062697
#36	Niacinamide	1.46	123.056	$[M+H]^+$	5	0.99	0.99	0.99	Pyridines and derivatives	Pyridinecarboxylic acids and derivatives	C00153	HMDB0001406
#37	N-Glycolylneuraminic acid	1.47	306.077	$[M-H-H_2O]^-$	3	0.11	0.98	0.32	Organooxygen compounds	Carbohydrates and carbohydrate conjugates	C03410	HMDB0000833
#38	Hypoxanthine	1.49	137.046	$[M+H]^+$	4	0.95	0.99	0.97	Imidazopyrimidines	Purines and purine derivatives	C00262	HMDB0000157
**#39**	**Adenosine monophosphate**	**1.65**	**346.055**	**$[M-H]^-$**	**3**	**0.94**	**0.96**	**0.95**	**Purine nucleotides**	**Purine ribonucleotides**	**C00020**	**HMDB0000045**
**#40**	**L-Leucine**	**1.69**	**132.102**	**$[M+H]^+$**	**3**	**0.10**	**0.92**	**0.31**	**Carboxylic acids and derivatives**	**Amino acids, peptides, and analogues**	**C00123**	**HMDB0000687**
**#41**	**L-Tyrosine**	**1.76**	**182.082**	**$[M+H]^+$**	**5**	**0.43**	**0.96**	**0.65**	**Carboxylic acids and derivatives**	**Amino acids, peptides, and analogues**	**C00082**	**HMDB0000158**
**#42**	**2'-Deoxyguanosine 5'-monophosphate**	**2.12**	**346.055**	**$[M-H]^-$**	**3**	**0.15**	**0.99**	**0.39**	**Purine nucleotides**	**Purine deoxyribonucleotides**	**C00362**	**HMDB0001044**
#43	Oxidized glutathione	2.24	611.145	$[M-H]^-$	5	0.80	0.95	0.87	Carboxylic acids and derivatives	Amino acids, peptides, and analogues	C00127	HMDB0003337
#44	L-Phenylalanine	3.72	164.072	$[M-H]^-$	3	0.48	0.73	0.59	Carboxylic acids and derivatives	Amino acids, peptides, and analogues	C00079	HMDB0000159
**#45**	**Guanosine monophosphate**	**3.74**	**362.051**	**$[M-H]^-$**	**3**	**0.61**	**1.00**	**0.78**	**Purine nucleotides**	**Purine ribonucleotides**	**C00144**	**HMDB0001397**
**#46**	**L-Phenylalanine**	**3.99**	**166.087**	**$[M+H]^+$**	**3**	**0.99**	**1.00**	**1.00**	**Carboxylic acids and derivatives**	**Amino acids, peptides, and analogues**	**C00079**	**HMDB0000159**
#47	L-Phenylalanine	3.99	120.081	$[M+H-H_2O]^+$	6	0.64	0.96	0.78	Benzene and substituted derivatives	Phenethylamines	C00079	HMDB0000159
**#48**	**Pantothenic acid**	**4.29**	**218.103**	**$[M-H]^-$**	**6**	**0.91**	**0.96**	**0.93**	**Organooxygen compounds**	**Alcohols and polyols**	**C00864**	**HMDB0000210**
**#49**	**L-Tryptophan**	**4.59**	**203.083**	**$[M-H]^-$**	**3**	**0.86**	**0.93**	**0.89**	**Indoles and derivatives**	**Indolyl carboxylic acids and derivatives**	**C00078**	**HMDB0000929**
#50	Bexarotene	6.92	331.206	$[M+H-H_2O]^+$	3	0.38	1.00	0.62	Prenol lipids	Retinoids	NA	HMDB0014452
#51	5-Heptyltetrahydro-2-oxo-3-furancarboxylic acid	7.93	227.130	$[M-H]^-$	4	0.70	0.89	0.79	Lactones	Gamma butyrolactones	NA	HMDB0030994
#52	**SP d16:0†**	**8.25**	**274.277**	**$[M+H]^+$**	**3**	**-**	**0.71**	**1.00**	**Organonitrogen compounds**	**Amines**	**NA**	**NA**
#53	*SP d16:0	8.29	230.248	-	-	-	-	-	Organonitrogen compounds	Amines	NA	NA
#54	Phytosphingosine	8.33	318.302	$[M+H]^+$	3	0.77	0.81	0.79	Organonitrogen compounds	Amines	C12144	HMDB0004610
#55	Isometheptene	8.90	142.160	$[M+H]^+$	6	0.82	0.88	0.85	Organonitrogen compounds	Amines	NA	HMDB0015651
#56	9-Deoxy-delta12-PGD2	9.07	395.244	$[M+CH_3COOH-H]^-$	4	0.04	1.00	0.21	Fatty Acyls	Eicosanoids	NA	HMDB0060103
#57	Sphinganine	9.13	302.307	$[M+H]^+$	3	0.51	0.52	0.51	Organonitrogen compounds	Amines	C00836	HMDB0000269
#58	(S)-2-Propylpiperidine	9.58	128.144	$[M+H]^+$	11	0.93	0.95	0.94	Alkaloids and derivatives	Alkaloids and derivatives	C06523	HMDB0030285
#59	Menthone 1,3-glyceryl ketal	10.08	227.165	$[M-H]^-$	4	0.92	0.98	0.95	Prenol lipids	Monoterpenoids	NA	HMDB0040004
#60	3-Nonyl-1H-pyrazole	10.90	98.097	$[M+2H]^{2+}$	3	0.18	0.77	0.37	Azoles	Pyrazoles	NA	HMDB0034210
#61	(E)-Calamene	11.54	422.384	$[2M+NH_4]^+$	5	0.03	0.70	0.14	Prenol lipids	Sesquiterpenoids	NA	HMDB0059910
#62	1-(5-Methyl-3-pyridinyl)-1-decanone	11.58	512.417	$[2M+NH_4]^+$	6	0.02	0.89	0.12	Carbonyl compounds	Ketones	NA	HMDB0031952
#63	Cholesta-4,6-dien-3-one	11.60	424.364	$[M+H+ACN]^+$	3	0.02	0.99	0.15	Steroids and steroid derivatives	Cholestane steroids	NA	HMDB0002394

Note: RT, retention time; dp, dot product; rdp, reverse dot product; †, annotated with LipidBlast. *: feature annotated as a fragment of an annotated metabolite with the same XCMS-CAMERA pcgroup (i.e., pseudospectrum), detected in the experimental and reference MS/MS spectrum (with m/z accuracy error <20 ppm and an intensity >50 AU and relative intensity >0.01% of the base peak). In bold, metabolites employed in the pathway analysis (Table 2 and Figure S5).

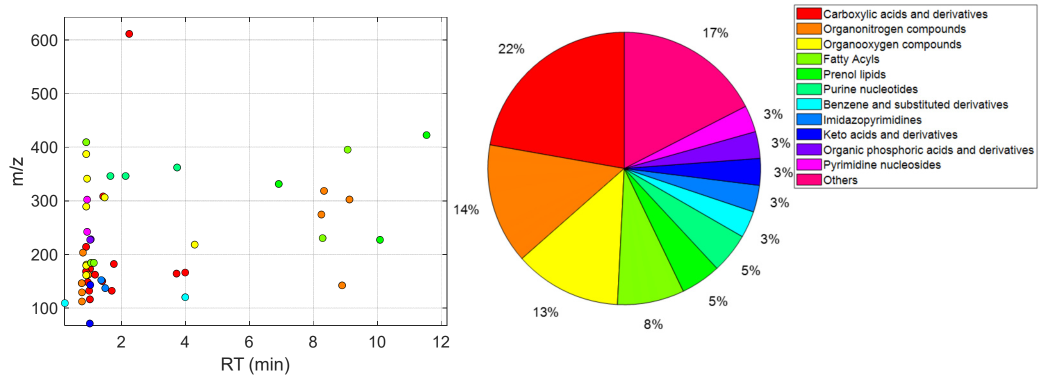

Figure S3. Annotated metabolic features in HM cells. Distribution of features in the m/z vs. retention time space (left) and proportion of annotated metabolite classes (right) detected in HM cells.

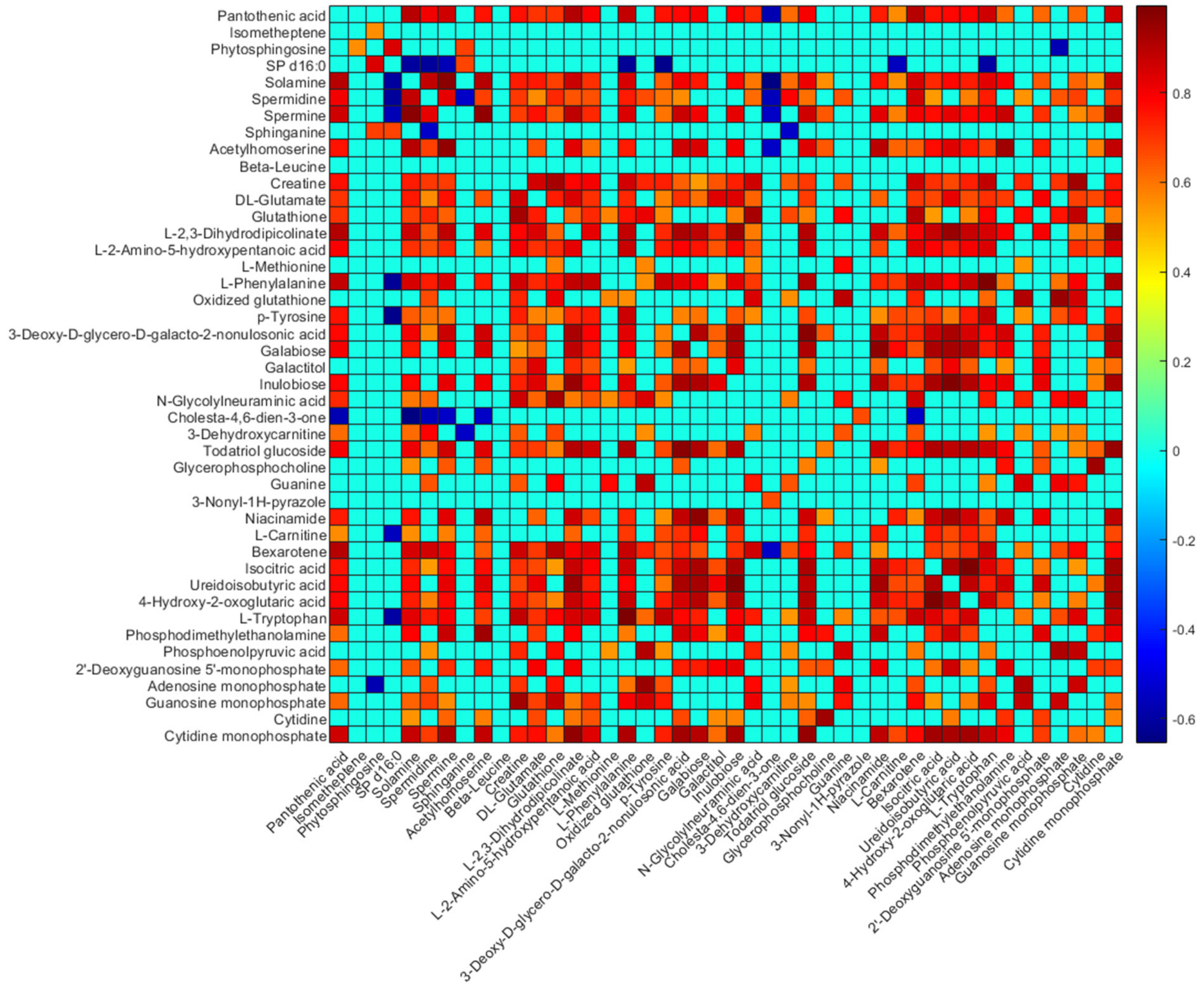

Figure S4. Pearson's paired correlation between unique metabolites. Note: For better visualization, those correlations considered non-significant (*p*-value < 0.05) are given an r-value = 0, and those metabolites that did not correlate significantly with any other metabolite are not shown.

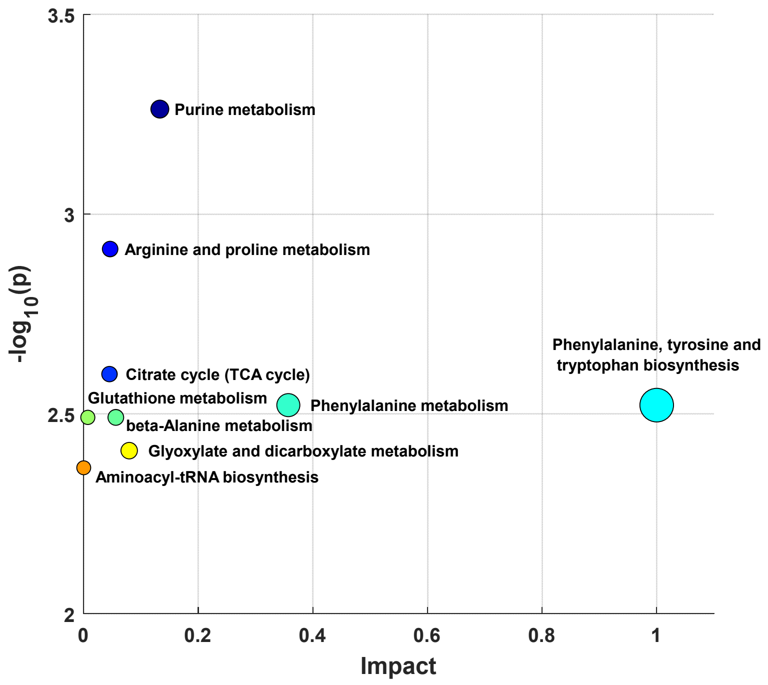

Figure S5. Pathway enrichment analysis results of metabolites correlating significantly with postnatal age.
